# A systematic structural comparison of all solved small proteins deposited in PDB. The effect of disulfide bonds in protein fold

**DOI:** 10.1016/j.csbj.2021.11.015

**Published:** 2021-11-17

**Authors:** Mariana H. Moreira, Fabio C.L. Almeida, Tatiana Domitrovic, Fernando L. Palhano

**Affiliations:** a Programa de Biologia Estrutural, Instituto de Bioquímica Médica Leopoldo de Meis, Universidade Federal do Rio de Janeiro, Rio de Janeiro, RJ 21941-902, Brazil; b Departamento de Virologia, Instituto de Microbiologia Paulo de Góes, Universidade Federal do Rio de Janeiro, Rio de Janeiro 21941-902, Brazil

**Keywords:** Protein fold, PDB, Disulfide bond

## Abstract

Defensins are small proteins, usually ranging from 3 to 6 kDa, amphipathic, disulfide-rich, and with a small or even absent hydrophobic core. Since a hydrophobic core is generally found in globular proteins that fold in an aqueous solvent, the peculiar fold of defensins can challenge tertiary protein structure predictors. We performed a Protein Data Bank survey of small proteins (3–6 kDa) to understand the similarities of defensins with other small disulfide-rich proteins. We found no differences when we compared defensins with non-defensins regarding the proportion of apolar, polar and charged residues and their exposure to the solvent. Then we divided all small proteins (3–6 kDa) in the Protein Data Bank into two groups, one group with at least one disulfide bond (bonded, defensins included) and another group without any disulfide bond (unbonded). The group of bonded proteins contained apolar residues more exposed to the solvent than the unbonded group. The *ab initio* algorithm for tertiary protein structure prediction Robetta was more accurate at predicting unbonded than bonded proteins. On the other hand, the trRosetta algorithm, which uses artificial intelligence, improved the prediction of most bonded proteins, while for the unbonded group no improvement was obtained. Our work highlights one more layer of complexity for the prediction of protein tertiary structure: The ability of small disulfide-rich proteins to fold even with a poorly hydrophobic core.

## Graphical abstract

**Figure:**
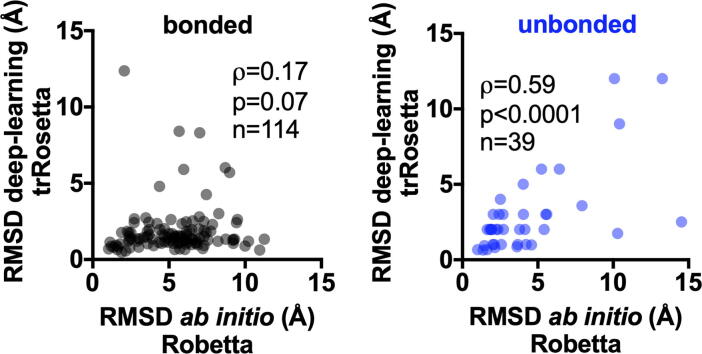

## 1 Introduction

Defensins are a group of small proteins (<10 kDa) related to host defense in animals, plants and fungi [1], [2], [3]. Their primary sequence is very diverse but rich in cysteines that form disulfide bonds [1], [2], [3], [4], [5]. The tertiary structure has a compact core, and they typically contain a triple-stranded antiparallel β-sheet, packed against an α-helix restrained by disulfide bonds [2]. Defensins frequently lack a hydrophobic core (thus forming a core-less fold), with a high proportion of hydrophobic residues exposed to the solvent [5], [6]. Other globular proteins form a hydrophobic core, which buries apolar residues, minimizing their solvent- accessible surface [7]. Almeida and collaborators solved the structure of sugarcane defensin 5 (SD5) [8], *Pisum sativum* defensin 1 (Psd1) [4] and *Pisum sativum* defensin 2 (Psd2) [9]. These proteins lack a hydrophobic core and present an unusual side-chain exposure of multiple hydrophobic amino acids [8], [9], [10]. The authors concluded that defensins are stabilized by tertiary contacts formed by hydrophobic surface clusters, solvent-stabilized clusters of surface-exposed hydrophilic and hydrophobic residues [8]. Remarkably, despite the multiple exposed hydrophobic residues, these proteins are highly soluble in water. It was hypothesized that the long polar/charged side chains of the surface clusters protect the hydrophobic amino acids from complete exposure to the solvent [9].

The features that allow the poor hydrophobic core of defensins to exist are still obscure, but it is likely that the presence of disulfide bonds plays an essential role in their fold. Even though it is well known that defensins possess a core-less fold, as far as we know, no systematic comparison of defensins with other small proteins containing or not disulfide bonds has yet been performed.

To address this issue, we analyzed all small protein structures (3–6 kDa) with at least one disulfide bond in the Protein Data Bank (PDB). The parameters we chose to compare defensins vs. non-defensin proteins were the percentage of apolar, polar and charged residues and the degree of their solvent exposure. No statistically significant differences were found between the two data sets, suggesting that besides defensins, other small proteins cross-linked by multiple disulfide bonds also display an unusual fold lacking a canonical hydrophobic core. Next, we compared all PDB small proteins (3–6 kDa) containing (bonded) or not (unbonded) disulfide bonds, following the same rationale. This time, we observed that the group with disulfide bonds has a lower proportion of apolar residues than the unbonded group. The apolar residues of the bonded group were more exposed to the solvent than those of the unbonded group, which compromised the formation of hydrophobic cores. We also compared the frequency and exposure of the long polar/charged side chains between the two groups, but no differences were found.

Prediction servers are now able to tackle challenging tertiary structures, resulting in satisfactory models [11], [12], [13], [14], [15], [16]. We challenge the *ab initio* and transform-restrained Rosetta (trRosetta) algorithms, available in the Robetta prediction server (http://robetta.bakerlab.org), which predicts tertiary protein structure, comparing the accuracy of prediction between bonded vs. unbonded peptides.

The models obtained by *ab initio* and trRosetta were compared with their respective PDB model from the RCSB PDB database. The structure comparison was measured by the root-mean-square deviation (RMSD) value, which the higher the value, the more deviated the obtained model is from the deposited PDB. Interestingly, the accuracy of the *ab initio* algorithm was greater for the unbonded peptides than for those with disulfide bonds, since the former possess the “canonical” hydrophobic core, while the trRosetta algorithm predicted well for both groups.

Our study indicates that even for small proteins, the *ab initio* prediction algorithms still have difficulty determining the protein structure, especially if the protein is cross-linked by disulfide bonds. Moreover, the new deep-learning algorithms circumvent this limitation and have high accuracy for predicting tertiary protein structure even in the absence of the canonical hydrophobic core.

## 2 Materials and methods

### 2.1 Data collection and analysis workflow

**Fig. 1:**
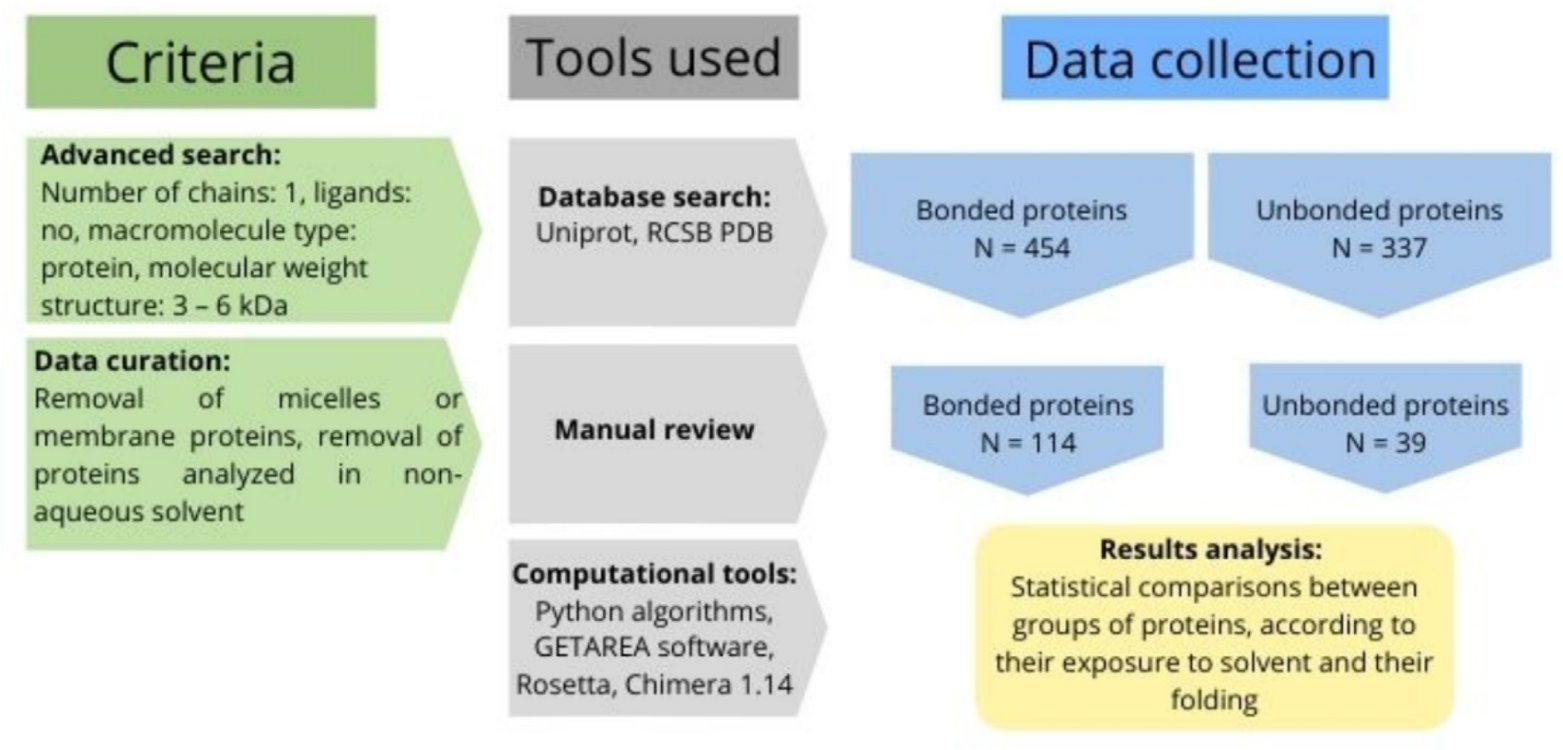
**Schematic representation of the pipeline used to obtain curated groups of bonded and unbonded small proteins.** After curating, 114 proteins for the bonded group and 39 proteins for the unbonded group were selected, as described in the text.

To understand how defensins compare to other small proteins, we chose the molecular weight ranging from 3 to 6 kDa because it allowed us to obtain a significant proportion of defensins in the bonded group (with disulfide bond). Fig. 1 shows a visual representation of the data collection and refinement that preceded the analysis of results. We performed an advanced search of PDB files in the RCSB PDB database and used the Uniprot database to collect FASTA sequences of the selected proteins. In advanced-search options of the RCSB PDB database, we could not refine the criteria sufficiently to obtain only proteins in an aqueous solvent, without ligands or other elements that could interfere in their folding, so we had to curate the data manually. After manual review, the number of proteins within the groups was significantly reduced because we aimed to obtain unbiased comparable groups of proteins without any variable that could interfere with their folding in an aqueous solvent. Bonded and unbonded groups are proteins with and without at least one disulfide bond, respectively.

**Fig. 2:**
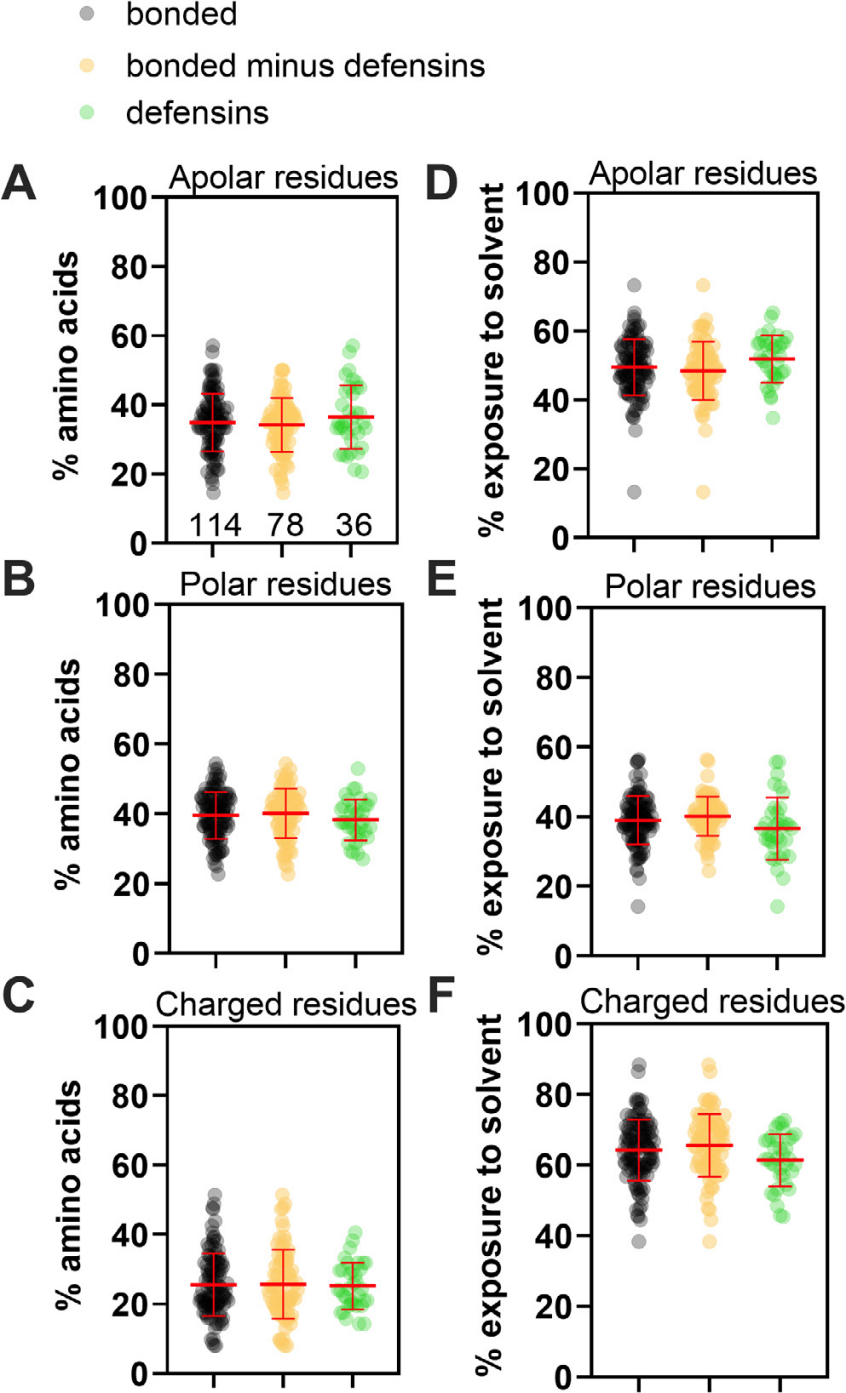
**Defensins present a similar percentage and solvent exposure of apolar, polar, and charged residues compared to other small disulfide-bonded proteins**. In all graphs, the whole population of bonded proteins is represented in black, N = 114; proteins of the bonded group without defensins are represented in orange, N = 78; and proteins of the group containing only defensins are represented in green, N = 36. The proportion of apolar (A), polar (B), or charged residues (C) was calculated for each protein. The residue’s average degree of solvent exposure was calculated using GETAREA (please see details in methodology). Exposure of apolar (D), polar (E), or charged (F) residues. Kruskal-Wallis test was performed for all comparisons, but no statistical differences were identified. Since no differences were observed, the following analyses involve only the bonded group, N = 114.

### 2.2 Protein selection

The RCSB PDB database available at rcsb.org was used as a source of 3D protein structures. Selected data were downloaded as a PDB file in October 2021, and subjected to the following query: Number of Protein Instances (Chains) per Assembly = 1 AND Molecular Weight per Deposited Model = [3 – 6] AND Disulfide Bond Count per Deposited Model = 0 AND Entry Polymer Types = “Protein (only)” AND Total Number of Non-polymer Instances = 0 AND Structure Keywords NOT CONTAINS WORDS “micelle, membrane, ligand”. In this first search, 337 structures were found. In a second advanced search, we selected the option “Disulfide Bond Count per Deposited Model” >= 1, and 454 proteins were found, so in this case, each protein had at least one disulfide bond in its structure.

Thus, two groups were formed, the bonded group containing proteins with at least one disulfide bond, and the unbonded group containing proteins without a disulfide bond. Before analyzing these data, both groups were subjected to manual curation. Only proteins in an aqueous solvent, with a well-defined secondary structure and following all the criteria described in advanced-search options were kept. Finally, the bonded group contained 114 proteins, and the unbonded group had 39 proteins. All data, including results, are available in Supplementary Table 1.

### 2.3 Solvent-accessible surface area (SASA) calculation

To calculate SASA for each protein, we subjected them to the software GETAREA [15], available online at curie.utmb.edu/getarea.html, and the software Chimera 1.14 [17], [18]. The SASA result obtained by GETAREA is given in percentual of residue exposure, so the calculation by Chimera had to be transformed into a percentage to be comparable with the other method.

GETAREA uses the intersection of half-spaces to find vertices of all intersecting atoms exposed to solvent [15]. Chimera 1.14 creates a molecular surface model using an embedded software from MSMS package [18], then analytical solvent-excluded and solvent-accessible surface areas per atom and residue are computed as “AreaSES” and “AreaSAS”, respectively.

After opening a PDB file in Chimera, the option “show” in “Surface” was selected from the “Action” menu. Then, using “Render by Attribute” from “Structure Analysis” in the “Tools” menu, the attributes “AreaSAS” and “AreaSES“ of residues were exported in a TXT file. These files were subjected to a Python software that we developed called ”SASA_chimera_calculation.py.“ This algorithm calculates for each residue its percentage exposure by Eq. (1):

(1) $$\mathrm{SASA}=\left[\frac{{\mathrm{AreaSAS}}_{r}}{\left({\mathrm{AreaSAS}}_{r}+{\mathrm{AreaSES}}_{r}\right)}\right].100\%$$

AreaSAS_r_ and AreaSES_r_ refer to each amino acid residue's values in Chimera's respective TXT files.

The percentage results obtained from both programs were subjected to further Python programs in order to calculate the mean values for apolar, polar and charged amino acid proportion and exposure in each protein.

### 2.4 Amino acid proportion and exposure calculations

An in-house Python software (percentage_proportion.py) was developed to process the results obtained by GETAREA and Chimera, and it classifies the amino acids according to Table 2. The percentage of apolar, polar, and charged amino acids present in the structure is calculated for each protein.

**Table 1:** Software and algorithms used for data acquisition.

**Software and algorithms**	**Source**	**Identifier**
GETAREA	Fraczkiewicz and Braun, 1998	
Chimera 1.14	Petterson at al., 2014; Sanner, Olson and Spehner, 1996	
Robetta	Yifan et al., 2013; Srivatsan et al., 2009	
SASA_chimera_calculation.py	This paper	github.com/mhoyerm/SASA_chimera_calculation
Percentage_proportion.py	This paper	github.com/mhoyerm/SASA_chimera_calculation
Percentage_exposure.py	This paper	github.com/mhoyerm/SASA_chimera_calculation
Amino-acid_proportion.py	This paper	github.com/mhoyerm/SASA_chimera_calculation
Amino-acid_exposure.py	This paper	github.com/mhoyerm/SASA_chimera_calculation

**Table 2:** Classification of amino acids according to polarity and charge.

**Classification**	**Amino acids**
Apolar	GLY, ALA, VAL, PRO, LEU, ILE, MET, TRP, PHE
Polar	CYS, SER, THR, TYR, ASN, GLN
Charged	LYS, ARG, HIS, ASP, GLU

Because the input files also contained SASA values for each amino acid, a Python software called percentage_exposure.py was used to calculate the average exposure of apolar, polar, and charged residues. For each group of classified amino acids, the arithmetic mean of their solvent exposure values is calculated. Thus, these Python software output files include percentage values for both amino acid proportion and average exposure of apolar, polar, and charged groups of amino acid residues present in the protein.

The software amino-acid_proportion.py and amino-acid_exposure.py analyzed the tendency to form surface clusters with polar or charged amino acids. These algorithms receive the same inputs as the previous, but in this case, the amino-acid classification follows Table 3 [9]. Like the algorithms described earlier the output files includes percentage values of amino-acid types and average exposure, respectively, for amino-acid residues classified as short-chain or long-chain present in the protein.

**Table 3:** Classification of polar and charged amino acids according to the length of side chain.

**Classification**	**Amino acids**
Short side chain	CYS, SER, ASN, ASP
Long side chain	THR, TYR, GLN, LYS, ARG, HIS, GLU
Non classified	GLY, ALA, VAL, PRO, LEU, ILE, MET, TRP, PHE

### 2.5 Ab initio 3D determination

The online server Robetta was used to predict our data's *ab initio* protein structures [19], [20]. Primary sequences were obtained from RCSB PDB or the database SwissProt from uniport.org. The primary protein sequence was submitted as a job for structure prediction in Robetta, with the option “AB only” selected. After determining the tertiary structure, the server provides five models, downloaded as a PDB file.

### 2.6 Deep learning-based modeling

Robetta also allows protein modeling through their deep learning-based prediction method, called trRosetta [16]. Similar to the procedure described for the *ab initio* determination of each protein structure, the primary sequence was processed on the Robetta server, but in this case, with the option “TR only” selected. The server provides 5 models for the tertiary structure that can be downloaded as a PDB file.

### 2.7 RMSD calculation

The root-mean-square deviation (RMSD) between the protein structure deposited on RCSB PDB and the protein structure obtained from Robetta (either by *ab initio* or the trRosetta method), was calculated by Chimera 1.14. Both structures were opened in Chimera, then in the menu “Tools”, the option “MatchMaker” from “Structure Comparison” was used to select a reference structure, which would be the one downloaded from RCSB PDB, and a structure to match, which would be the one obtained from Robetta.

In cases where the reference contained multiple structures, only the first was considered. It was combined with all five possible matches (because Robetta provides five structure models) to determine the lowest RMSD value. The option selected for chain pairing was “Best-aligning pair of chains between reference and match structure”. The alignment algorithm was Needleman-Wunsch, BLOSUM-62, 1 gap extension penalty. Options “include secondary structure score (30%)” and “compute secondary structure assignments” were selected. The option “iterate by pruning long atom pairs until no pair exceeds 2 angstroms” was chosen for matching.

### 2.8 Statistical analyses, correlation, and raw data

**Fig. 3:**
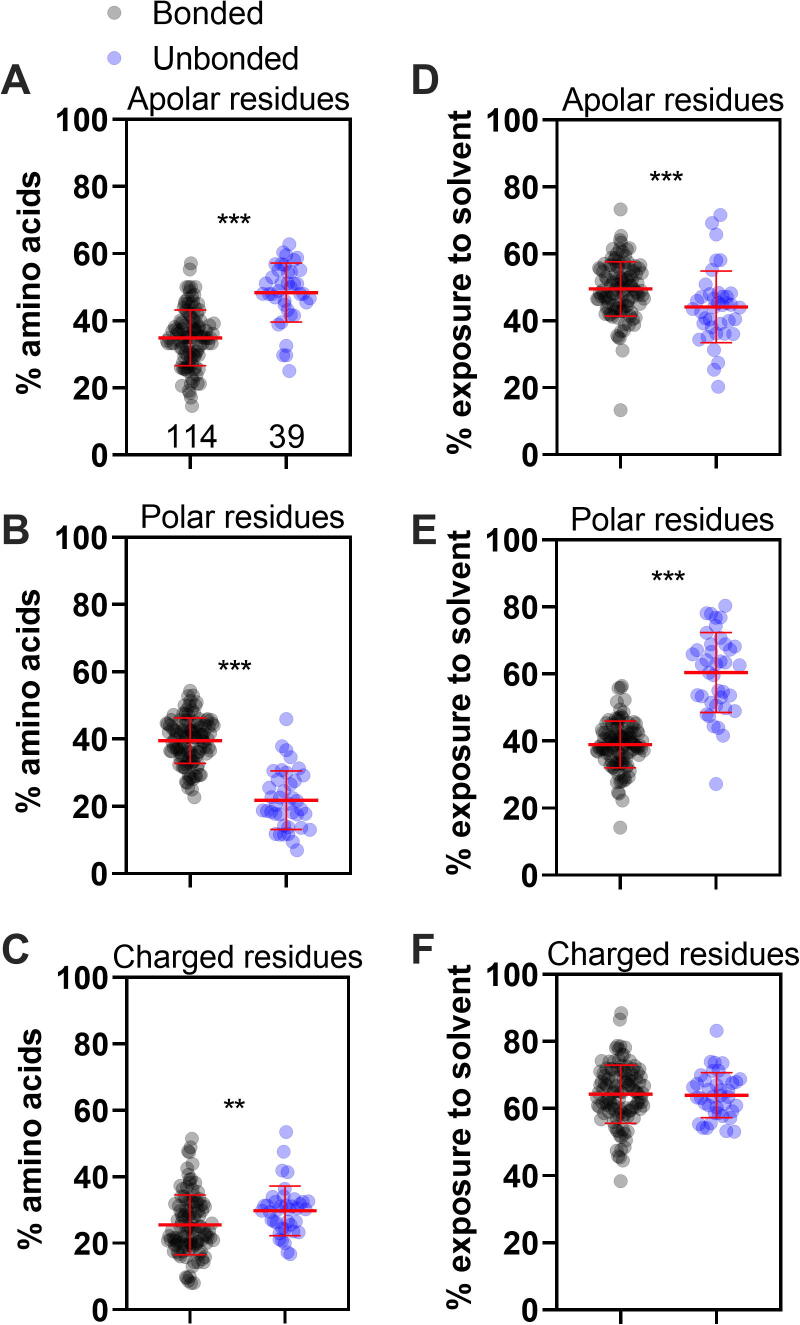
**Small proteins with at least one disulfide bond (bonded) present distinctive features regarding the proportion and exposure of residues compared to proteins that do not make disulfide bonds (unbonded)**. In all graphs, the whole population of bonded proteins is represented in black, N = 114, while the group of unbonded proteins is represented in blue, N = 39. The proportion of apolar (A), polar (B), or charged residues (C) was calculated for each protein. The average degree of solvent exposure was calculated using GETAREA. Exposure of apolar (D), polar (E), or charged (F) residues. Mann-Whitney test, **0.0037, ***<0.0001.

**Fig. 4:**
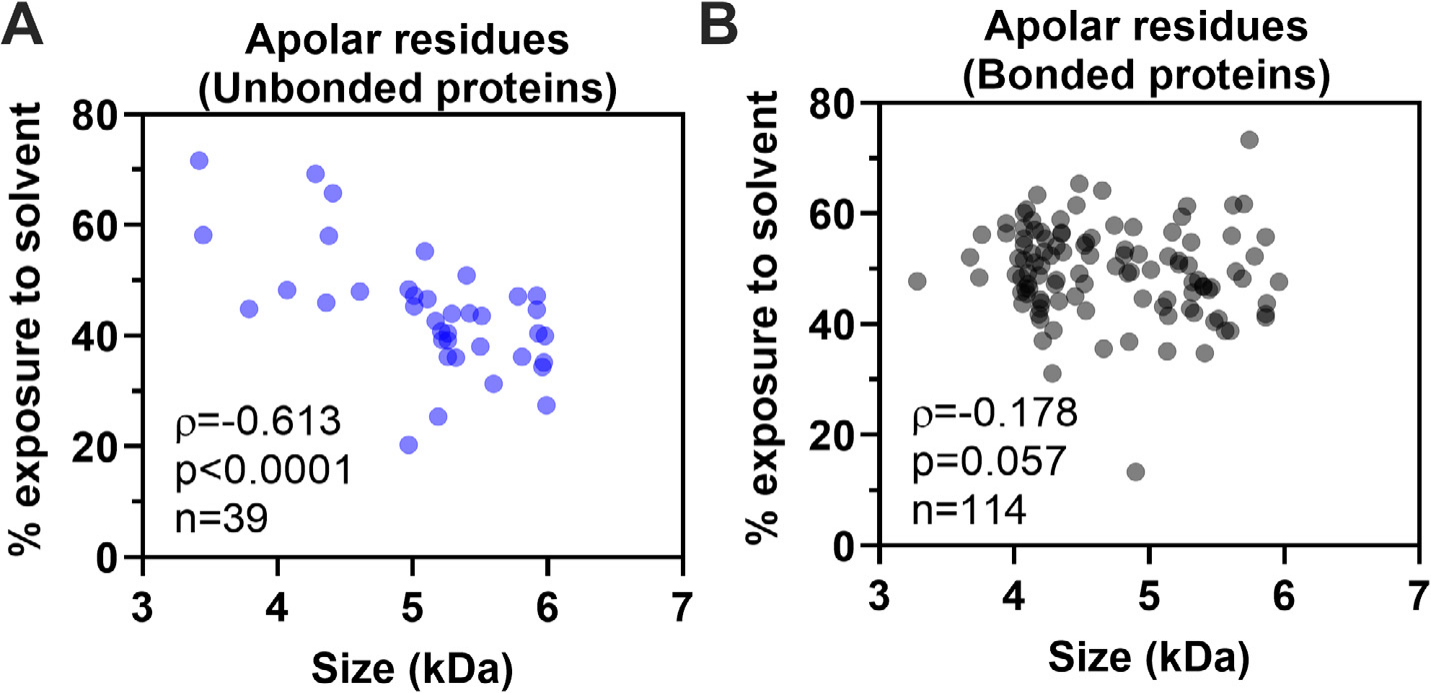
**The unbonded group presents a good correlation between protein size and exposure to solvent for apolar residues**. The Spearman correlation between apolar residues' exposure and protein size is negative for the unbonded group (A) and insignificant for the bonded group (B).

**Fig. 5:**
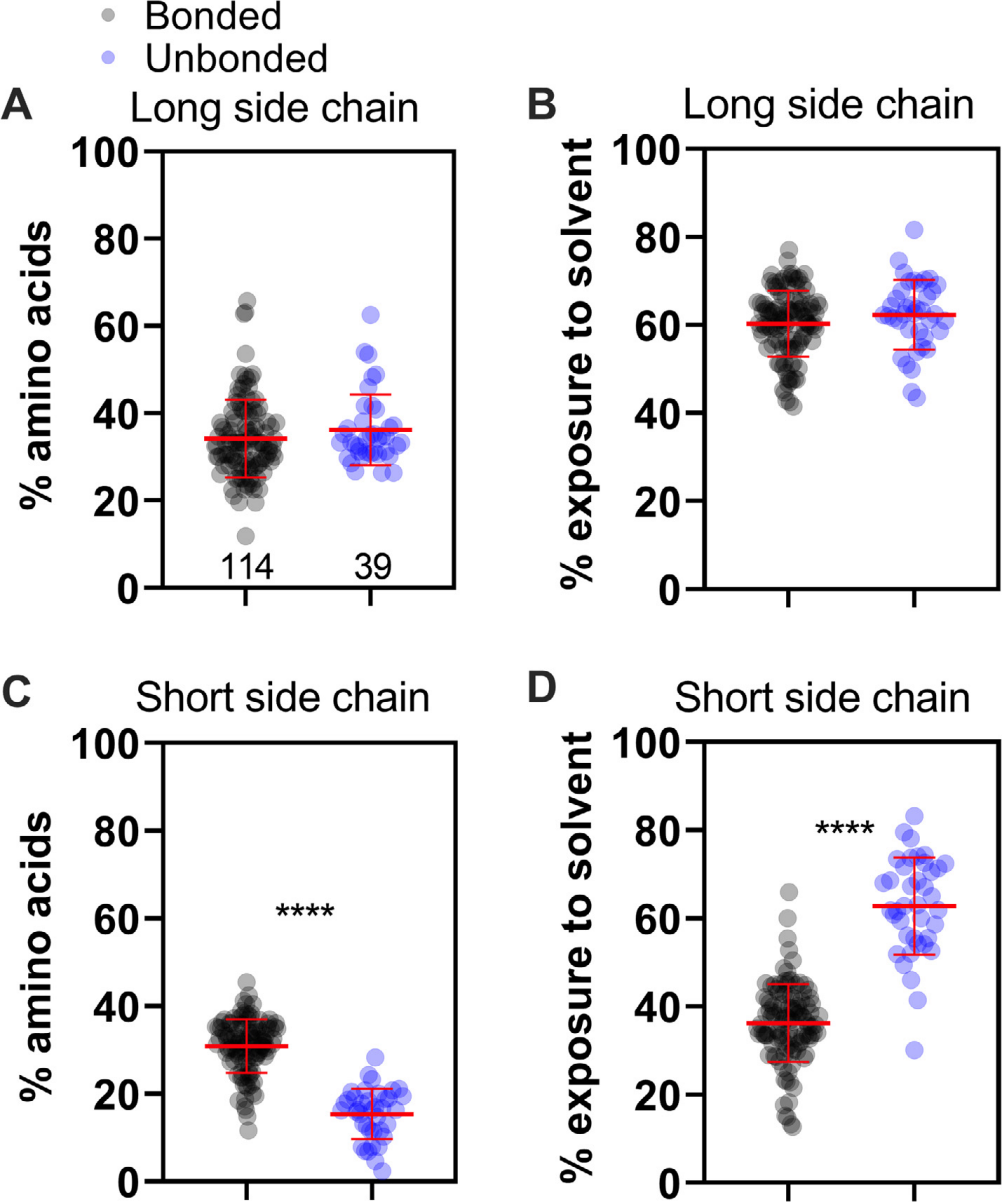
**Comparison between percentage and exposure of long or short side chains and polar/charged residues for the bonded and unbonded groups.** In all graphs, the bonded group proteins are represented in black, and proteins of the unbonded group are in blue. Comparison of the proportion (A) and solvent exposure (B) of long-chain polar/charged (A) residues among the bonded vs. unbonded groups of proteins. The same analysis was performed for short-chain polar/charged residues (C and D). Mann-Whitney test, ****<0.0001.

The raw data used to create all Figures in this paper is available in the Supplementary Table1. All statistical analyses were performed by GraphPad Prism 7. For Fig. 1, the Kruskal-Wallis test was used. For Fig. 3, Fig. 5, and Supplementary Fig. 1, the Mann-Whitney test was used. For Fig. 4, linear regression with a 95% confidence level was performed.

## 3 Results and discussion

### 3.1 Compared to other small cysteine-rich proteins, defensins do not appear to have an unusual fold to accommodate the poor hydrophobic core.

To understand how the tertiary structure of defensins differs from other small disulfide proteins, we downloaded PDB files from the RCSB PDB of all proteins with 3 to 6 kDa with at least one disulfide bond (bonded, 454 protein structures, Fig. 1). After manual review, the proteins solved in the presence of ligands, micelles, detergents, lipids, or organic solvents were eliminated, resulting in 114 proteins representing 25,1% of the initial sample (Fig. 1). The main classes of proteins in this group were defensins and toxins, with each class representing ∼ 30% of the total (Fig. S1). On average, each protein has 3 disulfide bonds and most structures (97%) were solved by NMR (Table S1). Using the software GETAREA we measured the proportion and degree of exposure to the solvent of all amino-acid side chains in these small proteins. First, we determined if, defensins have different amino acid composition from other proteins within the bonded group and thus should be treated separately in subsequent analysis. The bonded group (114 proteins) shown in Fig. 2 was divided in two subsets, one containing only defensins (36 proteins) and other containing non-defensins proteins (78 proteins). These groups were analyzed for parameters such as amino acid classification (apolar, polar, and charged, Table 2) in terms of proportion within the primary sequence (Fig. 2A-2C) and their exposure to the solvent (GETAREA analyses, Fig. 2D-F). Fig. 2 shows that there were insignificant differences among the three groups of proteins. We then decided to proceed with the entire bonded group, N = 114, for all subsequent analyses.

We conclude that if the defensins do present a core-less fold [8], [9], [21], their structure is not unique compared to other small disulfide proteins.

### 3.2 Small disulfide-rich proteins tend to expose more of their apolar residues to an aqueous solvent than non-disulfide proteins.

Since no differences were observed within the bonded group (Fig. 2), we then proceeded to analyze how the bonded group as a whole differs from small proteins without disulfide bonds (unbonded group) following the same pipeline described in Fig. 2. To define the unbonded group, we downloaded from the RCSB PDB the PDB files from proteins without disulfide bonds ranging from 3 to 6 kDa (337 protein structures). We followed the same manual review described for the bonded group, but, this time, we ended up with just 39 proteins (11,6 % of the initial sample). It is important to note that even starting with about 35% more proteins, after manual revision, the group of bonded proteins contained 3-fold more proteins than the unbonded group. One explanation for this discrepancy may be that small proteins without disulfide bonds are more dependent on cofactors to achieve their fold. Most of the proteins (87%) belonging to the unbonded group were solved by NMR (Table S1). Fig. 3 shows a comparison between bonded vs. unbonded groups according to amino acid classification (apolar, polar, and charged), in terms of proportion of each class within the primary sequence (Fig. 3A-C), and their exposure to solvent (GETAREA analyses, Fig. 3D-F). It is remarkable that essentially all parameters analyzed revealed significant differences between these groups. As observed in Fig. 3A and 3D, the bonded group, a lower proportion of apolar residues, can afford a greater degree of exposure to the aqueous solvent than the unbonded group. This result leads us to conclude that the bonded group of proteins does not present a canonical hydrophobic core in their structure. Even though it contradicts the expected folding model, it is consistent with what was observed for the structure of cysteine-rich peptides [21], [7], [8], [9].

Due to their tertiary structure stabilized by the disulfide bonds, proteins in the bonded group can afford this seemingly costly conformation. These proteins collapse in an enthalpically and entropically favorable ensemble, which allows the formation of disulfide bonds [6], [22]. However, their hydrophobic residues' exposure to the solvent makes these proteins more prone to form aggregates [6].

A similar conclusion was obtained on analyzing the area of solvent accessibility through the Chimera 1.14 software (Fig. S2). The result of Fig. 3F (GETAREA analyses) did not show a significant difference between groups regarding the exposure of charged residues. On the other hand, when the structures were analyzed by Chimera 1.14, we observed a slightly greater exposure of charged residues for the unbonded group compared to the bonded group (Fig. S2F).

We also evaluated whether exposing or hiding amino acid residues was correlated with protein size. Even though there is no consensus in the folding models for proteins with disulfide bonds, it is suggested that it is due to their disulfide bonds that these proteins have a stable structure [23]. Therefore, it is unlikely that the tertiary structure observed for this group of small proteins would be affected by the length of their amino-acid side chains. However, as the protein size increases, the folding pathway for the proteins of the unbonded group would be less affected by steric hindrance. Therefore, a canonical hydrophobic collapse would be more likely to occur. That being said, we compared the amount of exposure of apolar, polar, and charged residues vs. the protein size for both groups, unbonded and bonded (data not shown). Only the unbonded group has a negative correlation (ρ = −0.613) between protein size and exposure to solvent for apolar residues, as shown in Fig. 4.

The result of Fig. 4 reveals that even in a narrow size range (3 to 6 kDa), we could see the tendency for less exposure of the apolar residues as the protein size increases for proteins in the unbonded group. For example, the smallest protein in the group, PDB ID 6M56, has an average exposure of 72% for its apolar residues, while for the largest protein, PDB ID 2N8O, this exposure drops to 27%. It is important to note that the bonded group proteins may do not present their lowest free energy conformation as their native state, which is a possibility raised by Levinthal [24].

Pinheiro-Aguiar and colleagues noticed the presence of hydrophobic surface-clusters on defensin 2 from *Pisum sativum* [9]. The authors suggested that long polar/charged side chains in surface-clusters protect the hydrophobic amino acids from complete exposure to the solvent [9]. The following test was designed to determine whether the bonded group has some preferences for long polar/charged amino acids compared to the unbonded group. The amino acids were classified as short polar/charged (CYS, SER, ASN, ASP) or long polar/charged (THR, TYR, GLN, LYS, ARG, HIS, GLU, see also Table 3). The remaining amino acids (GLY, ALA, VAL, PRO, LEU, ILE, MET, TRP, PHE) were omitted in these analyses. First, the proportion in the primary sequence of long polar/charged residues was compared between bonded vs. unbonded groups. If the hypothesis of Pinheiro-Aguilar is correct, we expect to see a higher proportion of long polar/charged side chains in the bonded group, but it was not the case (Fig. 5A). Furthermore, no difference was found comparing the degree of solvent exposure of long polar/charged residues between the groups (Fig. 5B). On the other hand, we observed differences for both the proportion and degree of solvent exposure of short polar/charged residues between the groups (Fig. 5C-D). The bonded group presented a higher proportion and a lower exposure to the solvent for short polar/charged residues compared to the unbonded group (Fig. 5C-D). We conclude that no bias toward long polar/charged residues exists for the bonded group. It is unlikely that the model proposed by Pinheiro-Aguiar and colleagues explains the unusual solvent exposure of hydrophobic residues that occurs in the fold of defensins and other small disulfide-rich proteins. This work supports the participation of short polar side chains in the formation of the hydrophobic surface clusters.

### 3.3 Tertiary structures of small cysteine-rich proteins are better predicted by state-of-the-art algorithms.

**Fig. 6:**
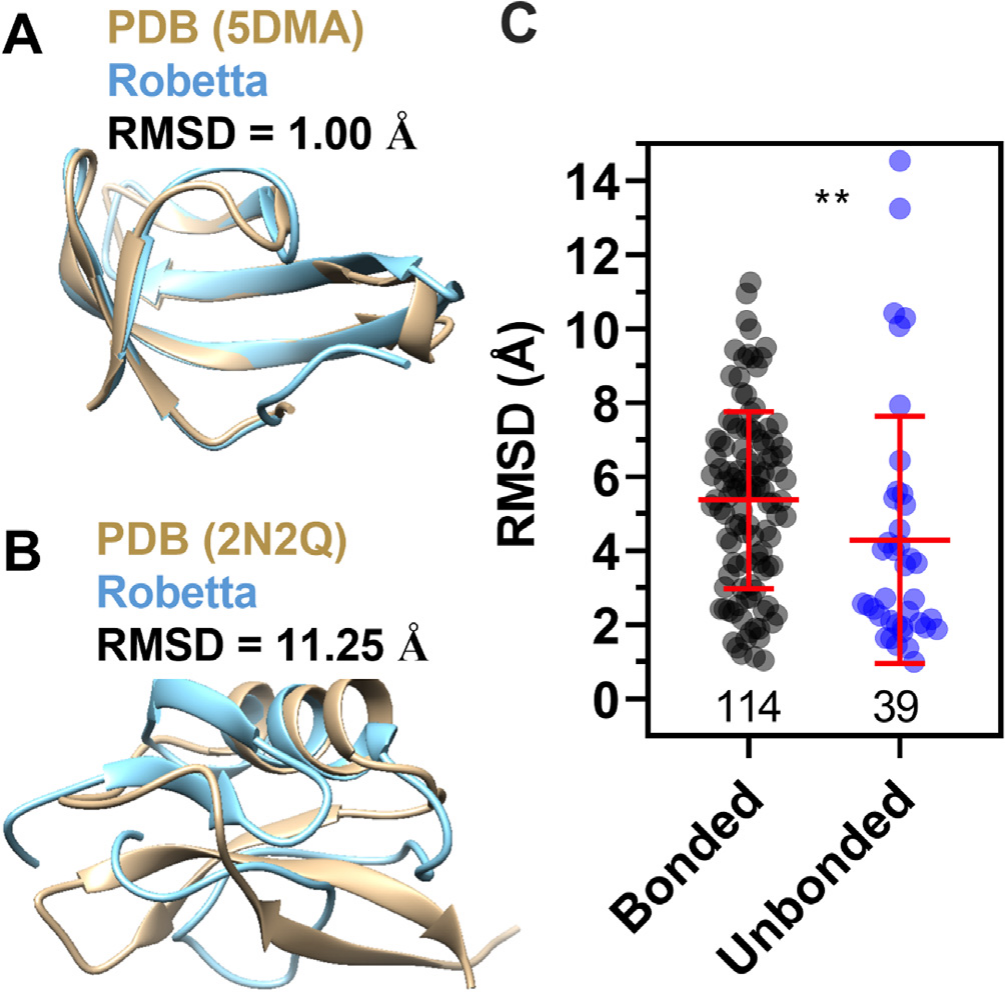
**The prediction accuracy of the *ab initio* algorithm Robetta was greater for the unbonded than for the bonded proteins.** Visual representation comparing original PDB files (in gold) for 5DMA (A) or 2N2Q (B) with the structures predicted by Robetta (in cyan). (C) Comparison between RMSD means for the bonded and unbonded groups. Mann-Whitney test, **0.0012.

**Fig. 7:**
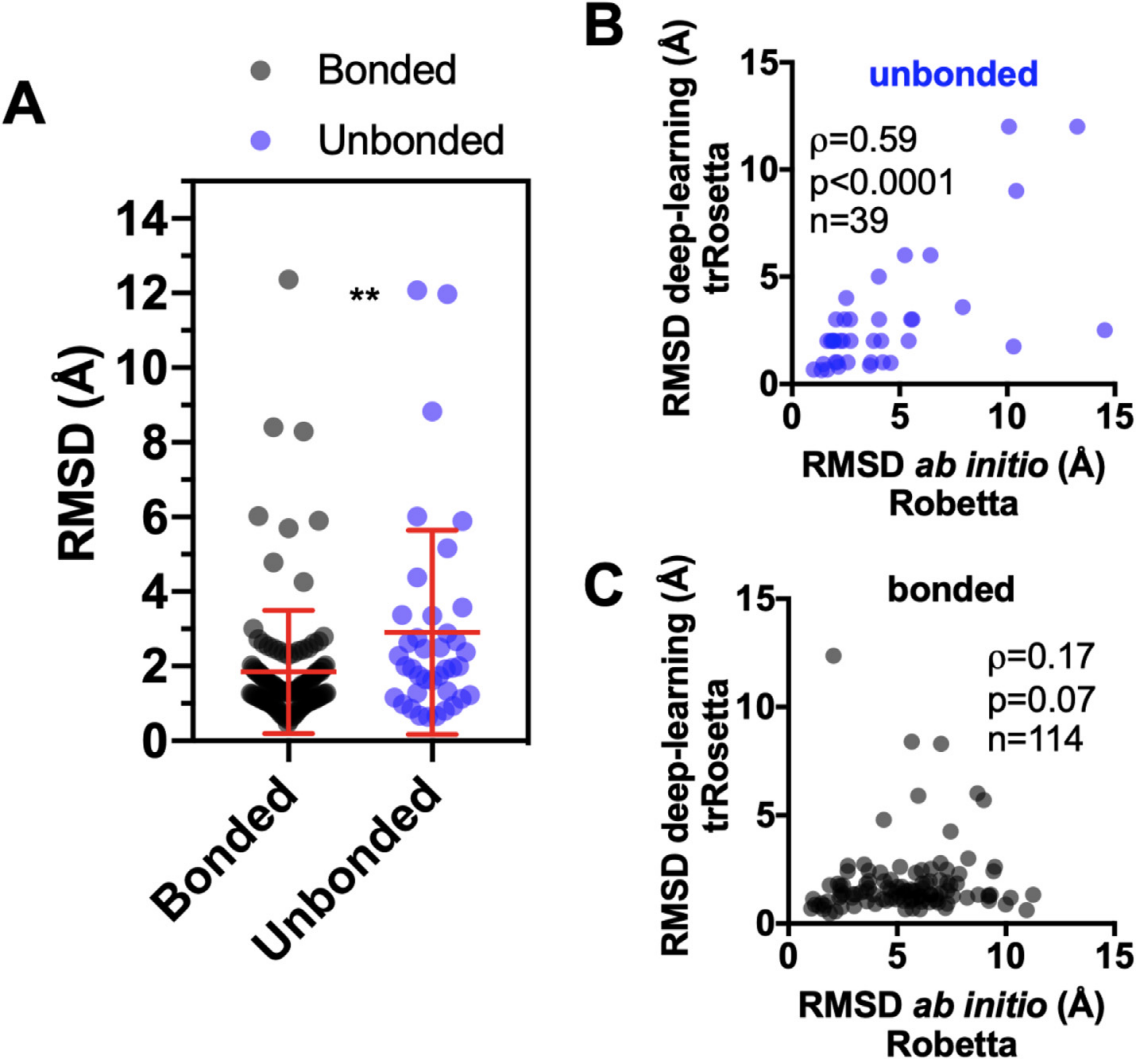
**trRosetta algorithm is more accurate in 3D protein structure prediction than the *abinitio* algorithm for the bonded group, but for proteins of the unbonded group the two methods do not differ.** Comparison between RMSD calculated for the bonded and unbonded groups using the algorithm trRosetta (A). Mann-Whitney test, p-value = 0.0042. A positive correlation between RMSD values obtained from *ab initio* (Robetta) vs. deep learning (trRosetta) methods was observed for the unbonded group (B) while no correlation exists for the bonded group (C).

To understand whether the structure of the bonded group proteins presents a non-obvious folding, we challenged the predictor server Robetta by selecting the *ab initio* option (Fig. 6) or the TR option (Fig. 7). We systematically submitted all proteins to the server because we wanted to compare how accurate the predictions are for both groups. Then, the structure obtained by Robetta was compared to the model in PDB file obtained from RCSB PDB, and the Chimera 1.14 software calculated the Root-mean-square deviation (RMSD) of atomic positions. Therefore, any difference observed between the groups' RMSD values is due solely to the predicted folding based on the proteins’ primary sequences.

Fig. 6A shows an example of accurate prediction: the original PDB (5DMA, gold) superposed well on the structure predicted by Robetta (cyan) with an RMSD of one angstrom. A different scenario was observed for the PDB 2N2Q, where the RMSD was eleven angstroms (Fig. 6B). Fig. 6C illustrates the comparison of RMSD values between the groups. We observed that the software Robetta predicted with greater accuracy the proteins belonging to the unbonded group (Fig. 6C). Finally, we correlated the proportion and degree of solvent exposure of apolar, polar, and charged residues with the RMSD. Again, we divided the proteins into two groups, unbonded and bonded and Fig. S3 shows the Spearman correlation for the mentioned parameters. We observed correlations with a p-value below 0.05 for two analyses, both for the unbonded group (Fig. S3B). The number of polar residues correlates positively with the RMSD (ρ = 0.43, Fig. S3C). The degree of solvent exposure of apolar residues also presented a positive correlation (ρ = 0.58) with RMSD. The Rosetta all-atom forcefield, used in *ab initio* prediction, is based on hydrogen bonding, short-range Van der Waals interactions, and desolvation [20], [25]. Although very realistic, this forcefield did not predict well the structure of our groups of peptides, especially those with disulfide bonds. This could indicate that the native conformation of the bonded group proteins is not their lowest free-energy state, and considering the extensive exposure of their apolar residues, this lack of hydrophobic core is probably not an evident folding. The structural prediction of small proteins is a challenging task [26], which is improving due to deep-learning techniques [27], as we were able to prove in the analyses using the TR option.

Fig. 7A illustrates the comparison of RMSD values between the groups, this time using the TR option in Robetta. Clearly, it is a better methodology for solving tertiary structures, due to smaller mean values of RMSD obtained for both groups when compared to the *ab initio* method (Fig. 6C). Surprisingly, however, the mean RMSD was higher for the unbonded group than for the bonded group using this methodology (Fig. 7A).

A positive correlation between RMSD values using deep-learning (trRosetta) vs. *ab initio* (Robetta) prediction was observed for the unbonded group, as shown in Fig. 7B. It indicates that for proteins in this group, both methodologies predict the tertiary structure with similar accuracy (Fig. 7B). On the other hand, no correlation was observed when the same analysis was performed for the proteins of the bonded group (Fig. 7C). For most proteins (105 among 114), the RMSD obtained from the deep-learning (trRosetta) algorithm was below 3 Å. A different scenario was observed when the *ab initio* (Robetta) method was used: few proteins (24 out of 114) presented RMSD equal to or under<3 Å (Fig. 7C).

We conclude that small disulfide proteins adopt an unusual fold without a hydrophobic core. However, due to improvements in deep-learning algorithms, the absence of a hydrophobic core does not compromise the accuracy of the tertiary structure predictors.

## Data availability

The algorithms used in this work are available in the GitHub repository (HTTPS:// github.com/mhoyerm).

## Funding

This work was supported by Conselho Nacional de Desenvolvimento Científico e Tecnológico (CNPq), Fundação de Amparo a Pesquisa do Estado do Rio de Janeiro (FAPERJ) e Coordenação de Aperfeiçoamento de Pessoal de Nível Superior (CAPES).

## CRediT authorship contribution statement

**Mariana H. Moreira:** Data curation, Formal analysis, Investigation, Methodology, Software, Validation, Visualization, Writing – original draft, Writing – review & editing. **Fabio C.L. Almeida:** Formal analysis, Investigation, Supervision, Writing – review & editing. **Tatiana Domitrovic:** Formal analysis, Investigation, Supervision, Writing – review & editing. **Fernando L. Palhano:** Conceptualization, Formal analysis, Funding acquisition, Investigation, Investigation, Project administration, Resources, Supervision, Visualization, Writing – review & editing.

## Declaration of Competing Interest

The authors declare that they have no known competing financial interests or personal relationships that could have appeared to influence the work reported in this paper.
