# Supplementary material for: A systematic structural comparison of all solved small proteins deposited in PDB. The effect of disulfide bonds in protein fold
Source: Comput Struct Biotechnol J. 2021 Nov 17;19:6255–62. doi: 10.1016/j.csbj.2021.11.015 (PMC8712280; doi:10.1016/j.csbj.2021.11.015)
Supplement: Supplementary data 1 [file mmc1.docx]

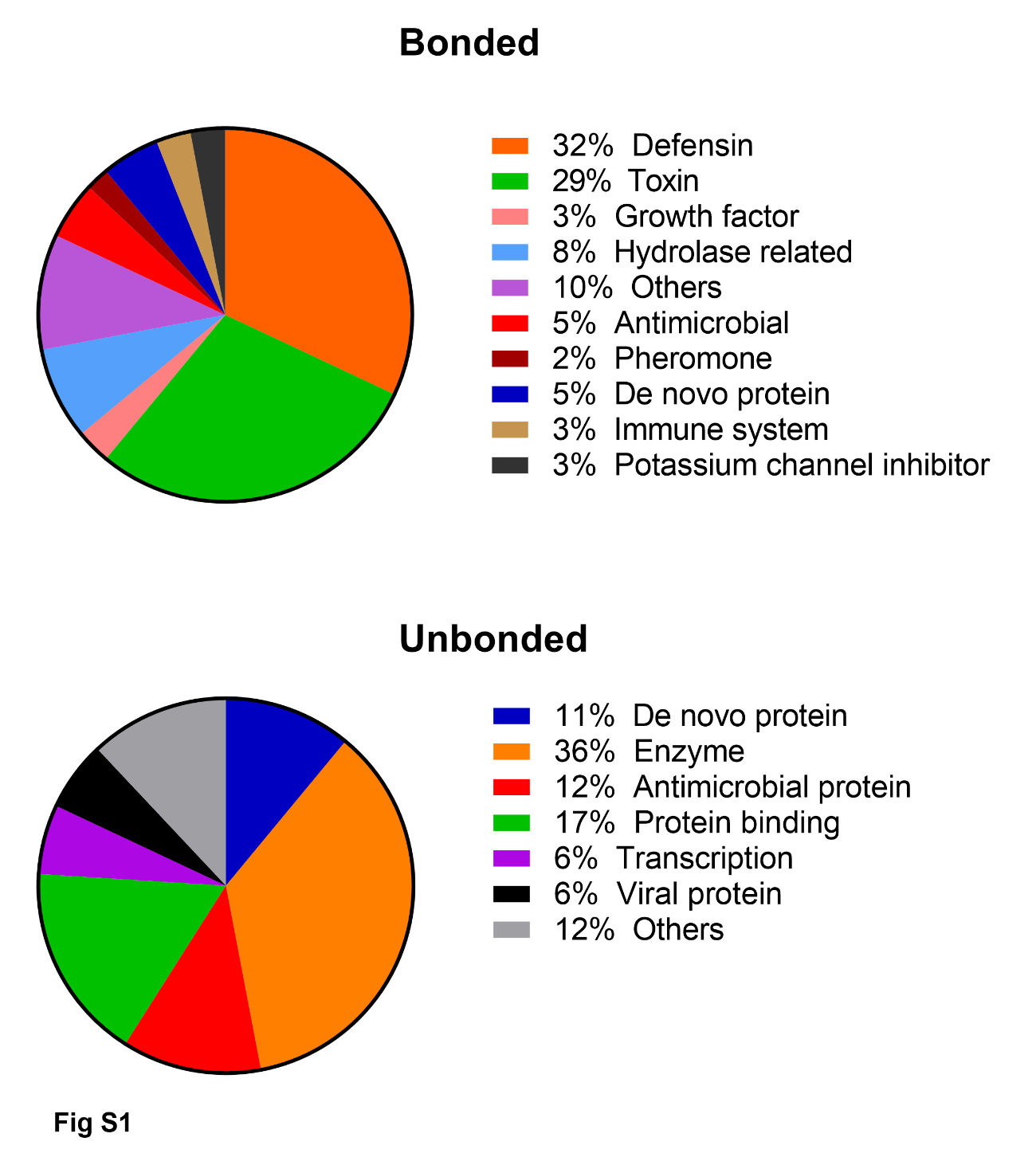


**Figure S1.** **Protein classification of the small proteins used in this study.** After manual curing we study 114 and 39 proteins in the bonded and unbonded groups respectively.


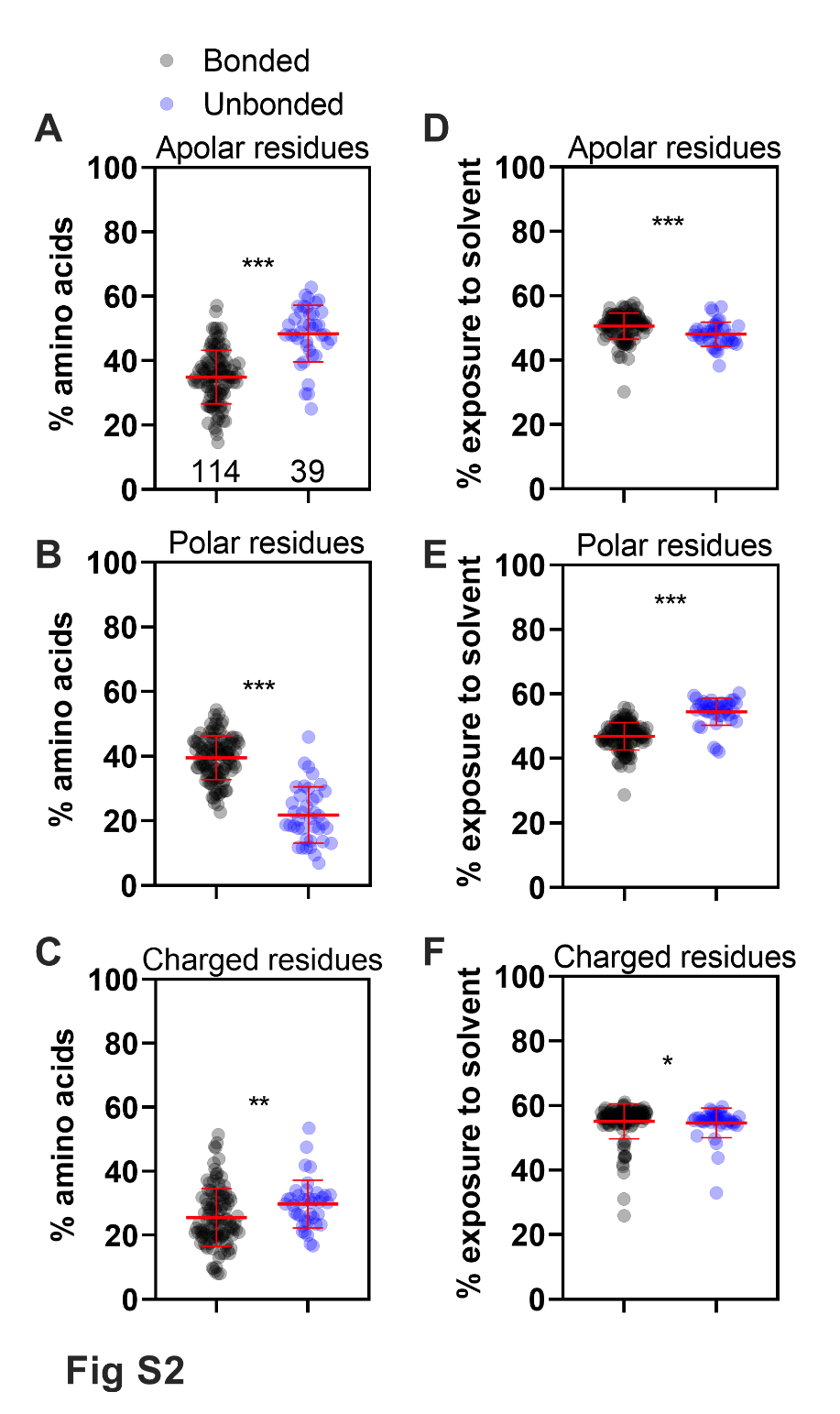


**Figure S2. Small proteins with at least one disulfide bond (bonded) present different features regarding the proportion and exposure of residues compared to proteins that do not form disulfide bonds (unbonded)**. In all graphs, the whole population of bonded proteins is represented in black, N=114, while the group of unbonded proteins is represented in blue, N=39. The proportion of apolar (A), polar (B), or charged residues (C) was calculated for each protein. The average degree of solvent exposure was calculated using Chimera. Exposure of apolar (D), polar (E), or charged (F) residues.


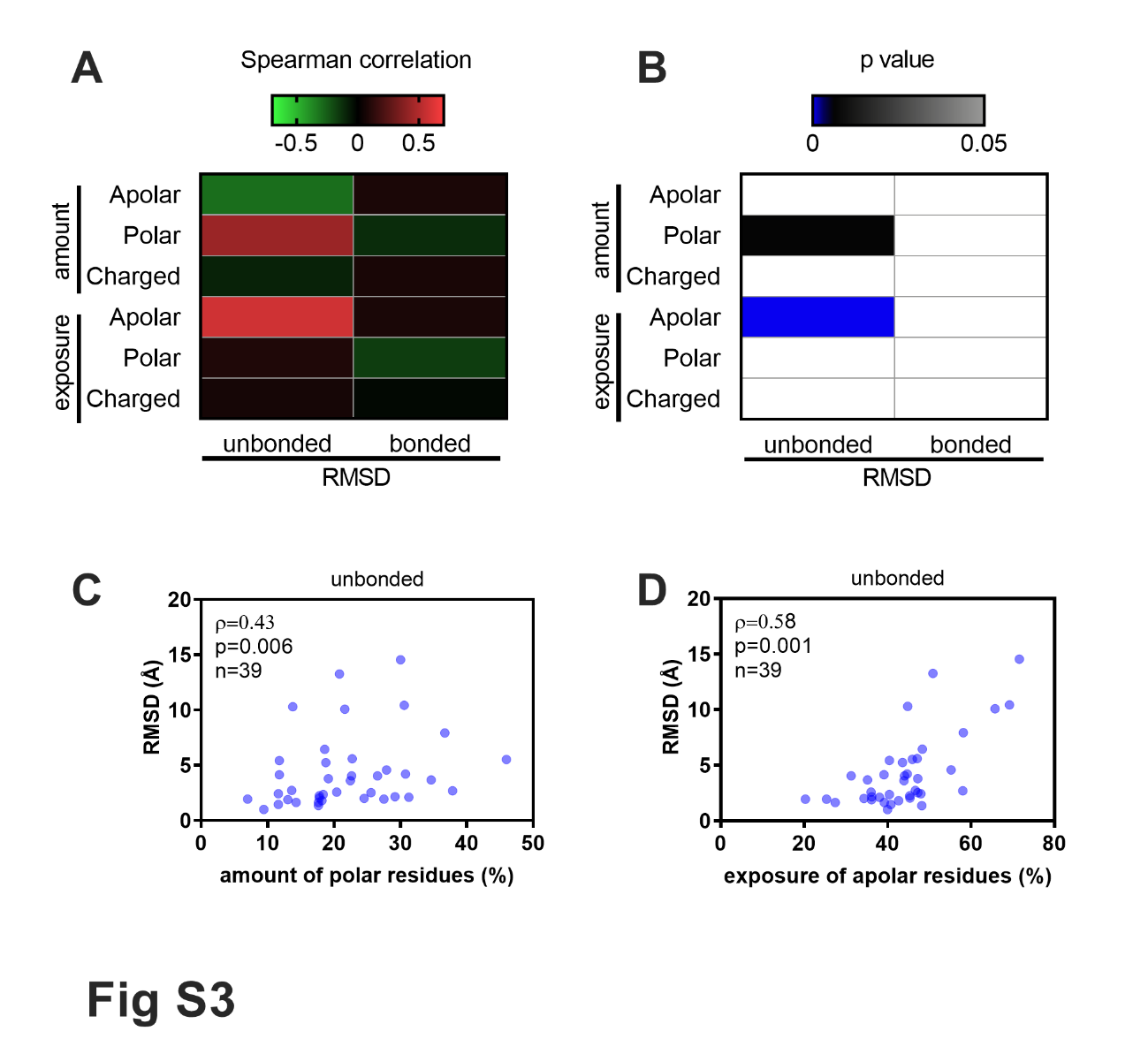


**Figure S3. Spearman correlation among the RMSD calculated from *ab initio* (Robetta) vs. the amount or exposure of amino acids**. The RMSD among the predicted structure calculated form *ab initio* (Robetta, see also Figure 6) was used to correlate with the proportion and exposure of apolar, polar and charged residues (se also Figure 3 of bonded and unbonded groups. The Spearman correlation and p value are showed in A and B respectively. In details the two parameters where statistical significances were found (C and D).
